# Dengue virus strain 2 capsid protein switches the annealing pathway and reduces intrinsic dynamics of the conserved 5’ untranslated region

**DOI:** 10.1080/15476286.2020.1860581

**Published:** 2021-01-07

**Authors:** Xin Ee Yong, Palur Venkata Raghuvamsi, Ganesh S. Anand, Thorsten Wohland, Kamal K. Sharma

**Affiliations:** a NUS Graduate School for integrative Sciences and Engineering Programme, National University of Singapore, Singapore; b Centre for Bioimaging Sciences, National University of Singapore, Singapore, Singapore; c Department of Biological Sciences, National University of Singapore, Singapore, Singapore

**Keywords:** RNA chaperone, FRET-FCS, genome circularization, RNA replication, kissing loop

## Abstract

The capsid protein of dengue virus strain 2 (DENV2C) promotes nucleic acid structural rearrangements using chaperone activity. However, the role of DENV2C during the interaction of RNA elements in the conserved 5’ untranslated region (5’UTR) to the 3’ untranslated region (3’UTR) is still unclear. Thus, we investigated the effect of DENV2C on the annealing mechanism of two RNA hairpin elements from the 5’UTR to their complementary sequences during (+)/(-) ds-RNAformation and (+) RNA circularization. DENV2C was found to switch the annealing pathway for RNA elements involved in (+)/(-) ds-RNA formation, but not for RNA elements related to (+) RNA circularization. In addition, we also determined that DENV2C modulates intrinsic dynamics and reduces kinetically trapped unfavourable conformations of the 5’UTR sequence. Thus, our results provide mechanistic insights by which DENV2C chaperones the interactions between RNA elements at the 5’ and 3’ ends during genome recombination, a prerequisite for DENV replication.

## Graphical Abstract

**Figure:**
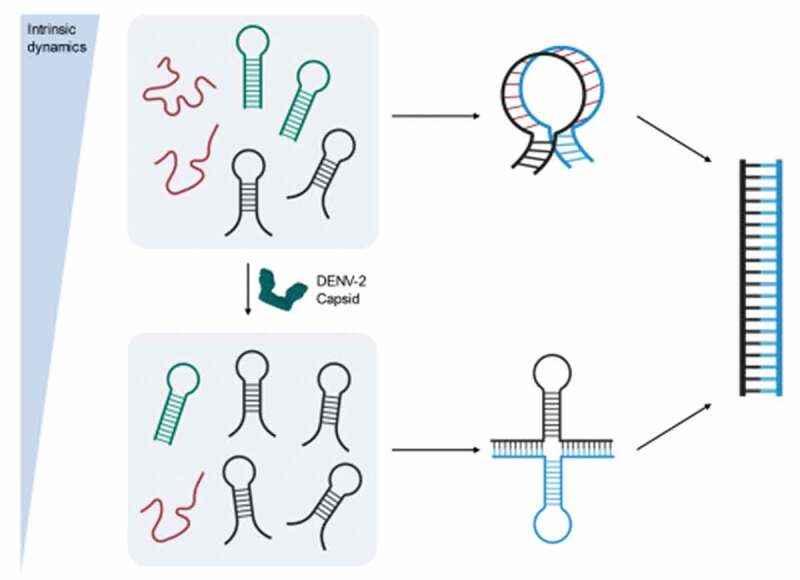

## Introduction

Dengue fever is caused by infection with dengue virus (DENV), which is transmitted by the bite of *Aedes aegypti* or *Aedes albopictus* mosquitoes carrying the virus. DENV has four antigenically-distinct serotypes, DENV1–4, which complicate vaccine development because an effective vaccine should neutralize all four serotypes effectively [1]. This is essential because secondary dengue infection tends to cause severe symptoms, such as dengue haemorrhagic fever and can even be fatal [2].

**Figure 1.**
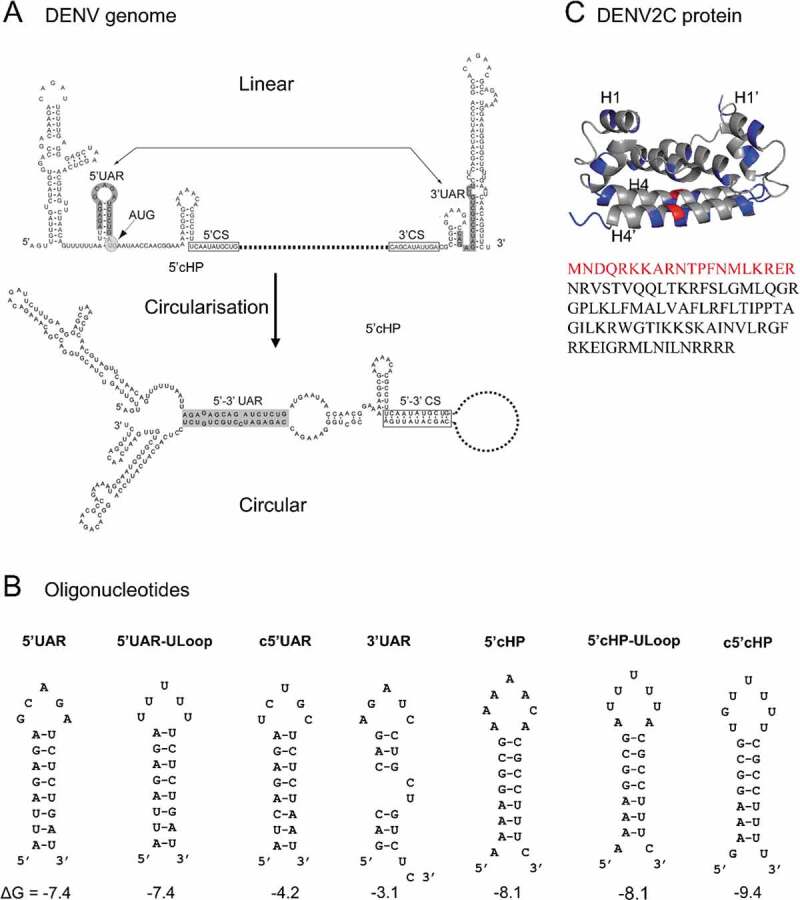
Structure of (A) DENV genome (adapted from [64]), (B) ORN sequences used in this study, and (C) DENV2C protein (PDB: 1R6R). (A) The 5ʹ and 3ʹ UTR region of DENV genome contain conserved circularization sequences, 5ʹ-3ʹ UAR (upstream AUG region) (grey). Annealing of these circularization sequences allow the formation of the circularized genome. (B) ORN sequences are derived from the 5ʹUAR, 5ʹcHP and 3ʹUAR region of the genome. Their secondary structures and ΔG values, shown below the structures in kcal/mol, were predicted using the mfold webtool (http://unafold.rna.albany.edu/). 5ʹUAR, 5ʹUAR-ULoop, 5ʹcHP and 5ʹcHP-ULoop are doubly labelled with 5ʹ FAM and 3ʹ TAMRA. Annealing pairs 5ʹUAR/c5ʹUAR and 5ʹcHP/c5ʹcHP represent (+)/(-) ds RNA, whereas 5ʹUAR/3ʹUAR are representing the (+) RNA circularization event. (C) The DENV2C protein is a homodimer. Blue and red residues highlight basic and acidic residues, respectively. The α1 helix (H1) and α4 helix (H4) are labelled for both subunits of the protein, with H1 and H4 from one subunit and H1ʹ and H4ʹ from the other subunit. In the protein sequence of DENV2C monomer, residues in red are part of the N-terminus disordered region which are not shown in the structure

DENV2 is a positive-sense single-stranded RNA (ssRNA) virus with an icosahedral structure (T = 3). The 50 nm virus has a genome of approximately 10.7 kb, which is translated to a polypeptide that is cleaved into three structural (envelope [E], pre-membrane [prM] and capsid [C]) and seven non-structural proteins (NS1, NS2A, NS2B, NS3, NS4A, NS4B, NS5) [3]. The coding region is flanked at both ends by untranslated regions (UTR). The 5′ end has a type I cap structure (m^7^GpppAmp) mediating cap-dependent translation, but the virus can switch to a noncanonical translation mechanism when translation factors are limiting [4]. Circularization of the (+) RNA genome is found to be essential for viral replication as mutations in conserved circularization RNA sequences reduces viral fitness [5,6]. This (+) RNA circularization of the DENV genome (Fig. 1A) is proposed to proceed either through protein-nucleic acid interactions [6] or via long range RNA-RNA-based 5′ and 3′ (5′-3′) end interactions, which can occur in the absence of proteins [7–12]. Thus far, two complementary RNA elements at the 5′ and 3′ ends have been identified for such long range 5ʹ-3ʹ RNA end interactions and are necessary for (+) RNA circularization [8]. One of these sequences, a hairpin structure, known as the 5′ upstream AUG region (5′UAR) element in the 5′UTR, anneals with its complementary 3′UAR counterpart, which is located at the bottom of 3′ stem loop (3’SL) in the 3ʹUTR (Fig. 1B) [7,9,12]. In addition to the RNA sequences involved in 5′-3′-end interactions necessary for the (+) RNA circularization, the 5′ end of the viral genome harbours another functional RNA element capsid-coding region hairpin (5ʹcHP). The 5ʹcHP element resides within the capsid-coding region and directs start codon selection for RNA translation [13,14]. It is believed that the 5ʹcHP stalls the scanning initiation complex over the first AUG, favouring its recognition [15] and stabilizing the overall 5′-3′ panhandle structure [13]. (+) RNA circularization is also essential for the synthesis of the DENV (-) RNA genome, which serves as template for the amplification of (+) RNA which can be packaged to synthesize new virions [12]. This (-) RNA genome synthesis includes NS5 binding at the 5′UTR and relocation of the polymerase at the 3′ initiation site via the (+) RNA circularization [8,16]. Thus, the hybridization of these complementary RNA elements at 5ʹ and 3ʹ ends are speculated to play a dual role during DENV transcription: (i) to bring the NS5 polymerase-5ʹUTR promoter complex near the 3′ end of the genome and (ii) to open the large stem of the 3′SL structure by 5′-3′UAR annealing [17]. Furthermore during (-) RNA genome synthesis, secondary structures of the conserved 5ʹUTR RNA elements, like 5ʹUAR and 5ʹcHP, which are templates for (-) RNA synthesis, melts and form double-stranded (+)/(-) RNA (ds-RNA) by annealing to their complementary (-) RNA sequences, c5ʹUAR and c5ʹcHP, respectively (Fig. S1A and C) [13]. The stem-loop structure of 5ʹUAR melts during both (+) RNA circularization as well as during (-) RNA synthesis, while the structure of 5ʹcHP melts only during (-) RNA synthesis, thus emphasizing the role of 5ʹUAR in stabilizing the circular over the linear form of DENV genome (Fig. 1A) [18].

Furthermore, both (+)/(-) ds-RNA formation and the (+) RNA circularization require the aid of RNA chaperones. RNA chaperones help to prevent misfolding of RNA by allowing RNA to sample many different conformations [19]. The DENV2 capsid protein, DENV2C, promotes annealing of the hammerhead ribozyme to its substrate, and promotes dissociation of the cleaved substrates, demonstrating RNA chaperoning ability [20]. DENV2C exists as a homodimer, consisting of two 100-aa subunits. The 13 kDa DENV2C is highly basic, containing 26/100 basic amino acids and has a flexible disordered region in the N-terminus, both key characteristics of many RNA chaperones (Fig. 1C) [21–24]. The protein associates with the ssRNA genome to form the nucleocapsid and encapsulates the nucleocapsid in a lipid bilayer containing E and M proteins [25]. DENV2C binds to RNA via its basic residues in helix-4 while binds to lipid bilayer using its hydrophobic cleft in helix-1 [26,27]. All further reference to DENV2C would be to one unit of the protein, i.e. the monomer.

Considering the abovementioned facts, DENV2C plays a vital role during virus formation possibly by modulating the interplay of RNA elements in the virus genome. Although the interaction of RNA elements in the DENV 5ʹ and 3ʹ ends is required for viral RNA replication, the role of DENV2C during this interplay is still not explored. We hypothesize that, due to its chaperone properties, the DENV2C is involved in genome rearrangements that are essential to RNA replication and viral fitness by modulating the annealing mechanism of the RNA element, 5ʹUAR, during (+)/(-) ds RNA formation and (+) RNA circularization.

To achieve this, we characterized the role of DENV2C as an RNA chaperone by investigating the annealing kinetics of 21-nt 5ʹUAR and 23-nt 5ʹcHP to their complementary sequences in the context of (+)/(-) ds-RNA formation and (+) RNA circularization. To represent the (-) RNA synthesis or (+)/(-) ds-RNA formation, we investigated annealing kinetics of the 21-nt long 5ʹUAR and 23-nt long 5ʹcHP with their complementary c5ʹUAR and c5ʹcHP, respectively (Fig. 1B). On the other hand, the (+) RNA circularization event is modelled by the annealing of 21-nt long 5ʹUAR with its complementary 3′UAR counterpart (Fig. 1B), which is located at the bottom part of 3′SL of the viral RNA. We doubly labelled 5ʹUAR and 5ʹcHP with 6-carboxyfluorescein (FAM) as the donor fluorophore at the 5ʹ end and carboxytetramethylrhodamine (TAMRA) as the acceptor fluorophore at the 3ʹ end, forming a Förster resonance energy transfer (FRET)-pair. Due to the proximity of donor and acceptor dyes in hairpin conformation of both 5ʹUAR and 5ʹcHP, the fluorescence of FAM is quenched. With the addition of a complementary sequence, the hairpin converts into a double-stranded sequence (extended duplex), in turn increasing the distance between donor and acceptor fluorophores. This increase in distance leads to florescence recovery of the donor that will provide information about the real-time annealing kinetics. By monitoring the annealing kinetics of the native and mutated 5ʹUAR and 5ʹcHP, we found that DENV2C promotes all three 5ʹUAR/c5ʹUAR, 5ʹcHP/c5ʹcHP and 5ʹUAR/3ʹUAR annealing. Interestingly, DENV2C is also able to switch the 5ʹUAR/c5ʹUAR annealing pathway that predominantly nucleates via the hairpin loops to a reaction pathway, nucleating through the hairpin stems. However, DENV2C does not alter the 5ʹUAR/3ʹUAR annealing pathway. We also determined by using FRET-fluorescence correlation spectroscopy (FRET-FCS) and time-resolved FRET (trFRET) that DENV2C exerts its chaperone functioning by reducing intrinsic dynamics of the 5ʹUAR and probably favouring one of the active conformations of the RNA hairpin. We also proposed mechanisms for 5ʹUAR/c5ʹUAR and 5ʹUAR/3ʹUAR annealing and compared the effects of DENV2C on (+)/(-) ds-RNA formation and (+) RNA circularization. Overall, our results provide the reaction mechanism by which DENV2C modulates the annealing of the 5ʹUAR sequence during (+)/(-) ds RNA formation and (+) RNA circularization, revealing how DENV2C can play a role in genomic rearrangements of 5ʹUAR that are essential to RNA replication and DENV fitness.

## Materials and methods

### Oligonucleotides

All oligoribonucleotides (ORN) were synthesized by Integrated DNA Technologies (Singapore). The doubly labelled ORNs (5ʹUAR, 5ʹUAR-ULoop, 5ʹcHP and 5ʹcHP-ULoop) were synthesized with 6-carboxyfluorescein (FAM) at the 5ʹ end and carboxytetramethylrhodamine (TAM) at the 3ʹ end. All ORNs were purified by the manufacturer using HPLC.

### DENV2C protein synthesis and purification

pET-21a(+) vector containing DENV2C protein gene sequence with N-terminal His tag and Tobacco Etch Virus (TEV) digestion site was purchased from GenScript (China). Recombinant capsid protein from DENV2 NGC strain was expressed in *Escherichia coli* BL21 strain. Transformed cells were grown in Luria-Bertani (LB) media supplemented with ampicillin (100 μg/ml) at 37°C until an optical density, OD_600_ of 0.6–0.8 was reached. A final concentration of 1 mM of isopropyl β-D-1-thiogalactopyranoside (IPTG) was used to induce expression and incubated for 16 to 18 hours at 18°C. This overnight culture was harvested by centrifugation at 6000 x g for 15 minutes at 4°C. The cells were lysed by sonication in NTE buffer consisting of 50 mM Tris-HCl, 1 M NaCl, pH 7.5 with 0.8 mM DTT, protease inhibitor cocktail (COEDTAF-RO, Merck) and 40 μg/ml of RNase A (Thermo Fisher Scientific). The cell pellet was separated by centrifugation at 13,000 rpm for 30 minutes, and the supernatant was collected and incubated with Cobalt beads for affinity chromatography. DENV2C protein was eluted at 1 M imidazole in NTE buffer and the collected elute was subjected to size exclusion chromatography (HiLoad 16/600 Superdex 200, GE Healthcare). The fraction containing DENV2C protein were pooled and dialysed against 50 mM HEPES, 150 mM NaCl, pH 7.5 buffer to remove imidazole and reduce NaCl concentration. Dialysed DENV2C protein was concentrated using Amicon Ultra-4 Centrifugal Filter Units (10 kDa molecular weight cut off, Millipore). The concentration of the purified protein sample was determined using nanodrop. DENV2C protein preparations were ribonuclease-free (Fig. S2) and RNA-free, determined by a A260/A280 value of 0.7 for the purified protein, very close to the theoretical value of 0.57 for a protein sample not contaminated by nucleic acids.

### Fluorescence spectroscopy

Fluorescence spectroscopy measurements were done on Cary Eclipse Fluorescence Spectrophotometer (Agilent) with a temperature control module, using Hellma® fluorescence cuvettes with an internal chamber volume of 45 μL. Excitation and emission wavelengths of 480 nm and 520 nm were used to track the intensity of 5ʹ FAM on doubly labelled ORNs in real-time. Reactions were done in pseudo first order conditions, with the concentration of non-labelled ORN being at least 10-fold more than the doubly labelled ORN. Equal volumes of both reactants were mixed at the start of the reaction to prevent high local concentrations of either reactant. For reactions in the presence of DENV2C, the protein was added to each ORN and mixed well before the two reactants were mixed. DENV2C:ORN ratios of 2:1 was used for all reactions in the presence of DENV2C protein. All reactions were done in 50 mM HEPES, 30 mM NaCl, 0.2 mM MgCl_2_, pH 7.5 buffer and at 20°C, unless otherwise stated. Temperature-dependence experiments to obtain Arrhenius parameters were done by equilibrating both reactants at the specified temperature for 10 minutes prior to mixing. All curve fitting was done on OriginProTM software (ver 9.55).

### FCS and FRET-FCS

FCS measurements were carried out on a commercial Olympus FV1200 laser scanning confocal microscope (Olympus, Singapore) equipped with an FCS upgrade kit (PicoQuant, Berlin, Germany). Doubly labelled ORN samples were excited with a 543 nm continuous wave laser. The beam was focused onto the sample by a water immersion objective (60×, NA 1.2; Olympus, Singapore) after being reflected by a dichroic mirror (DM405/485/543/635, Olympus, Singapore) and the scanning unit. The 3ʹTAMRA fluorescence from 5ʹUAR was recorded by a single molecule avalanche photodiode (SPAD) (SPCM-AQR-14, PerkinElmer Optoelectronics, Quebec, Canada), through a 600/50 band pass emission filter. Detected photon counts are registered by a TimeHarp 260 time-correlated single photon counting board (PicoQuant). Autocorrelation analysis was done using SymPhoTime 64 (PicoQuant, Berlin, Germany) to obtain diffusion time and number of fluorescent particles. ORN and DENV2C protein mixtures were incubated for 10 minutes before measurements. All measurements were performed at room temperature.

For FRET-FCS, the fluorescence signal from doubly-labelled 5ʹUAR was spectrally divided into donor and acceptor channels by a 560 dichroic longpass (DCLP) mirror. The donor and acceptor fluorescence were recorded using a set of single-molecule avalanche photodiodes (SPADs) (SPCM-AQR-14, PerkinElmer Optoelectronics, Quebec, Canada), through a 513/17 and 615/45 band pass emission filter (Omega, VT), respectively. The intensities of the donor and acceptor channel are collated in 20 μs time bins in the SymPhoTime64 software and exported. Using a home-written MATLAB script, the proximity ratio was calculated for each time bin and the autocorrelation of the proximity ratio was calculated. The calculated autocorrelation of the proximity ratio was then fitted using the Levenberg-Marquardt iteration algorithm by Origin 9.1.

### Time-resolved FRET (trFRET)

trFRET measurements were carried out on the same commercial Olympus FV1200 laser scanning confocal microscope equipped with a time-resolved LSM upgrade kit (Microtime 200, PicoQuant, GmbH, Berlin, Germany). Doubly labelled 5ʹUAR ORN was excited with a 485 nm pulsed diode laser with a 20 MHz repetition rate and 29 mW power (PDL series, Sepia II combiner module). The beam was focused into the sample by a water immersion objective (60×, NA 1.2; Olympus, Singapore) after being reflected by a dichroic mirror (DM405/485/543/635, Olympus, Singapore) and the scanning unit. The fluorescence was collected by the same objective followed by a pinhole (120 mm) to remove out-of-focus light. The fluorescence signal was spectrally divided into donor (green) and acceptor (red) channels by a 560 DCLP mirror. The 5ʹFAM donor fluorescence was recorded by a SPAD (SPCM-AQR-14, PerkinElmer Optoelectronics, Quebec, Canada), through a 513/17 band pass emission filter (Omega, VT). This donor signal was further processed by a time correlated single photon counting card (TimeHarp 260, PicoQuant) to build up the histogram of photon arrival times. The trFRET measurements were recorded for 180 s after incubating 5ʹUAR and DENV2C samples for 10 min at room temperature. The mean lifetime (τ) was calculated from the individual fluorescence lifetimes (τ_i_) and their relative amplitudes (a_i_) according to $\left(\tau \right)=\sum^{\alpha_{i}}\tau_{i}$. Donor fluorescence lifetime decay data were treated using the software SymPhoTime 64 (PicoQuant, GmbH). In all cases, the $\chi^{2}$ values were close to 1 and the weighted residuals as well as their autocorrelation were distributed randomly around 0, indicating a good fit. The reported values are mean and S.D.’s from at least three replicates.

## Results

### 5ʹUAR/c5ʹUAR and 5ʹcHP/c5ʹcHP annealing is faster than 5ʹUAR/3ʹUAR annealing

**Figure 2.**
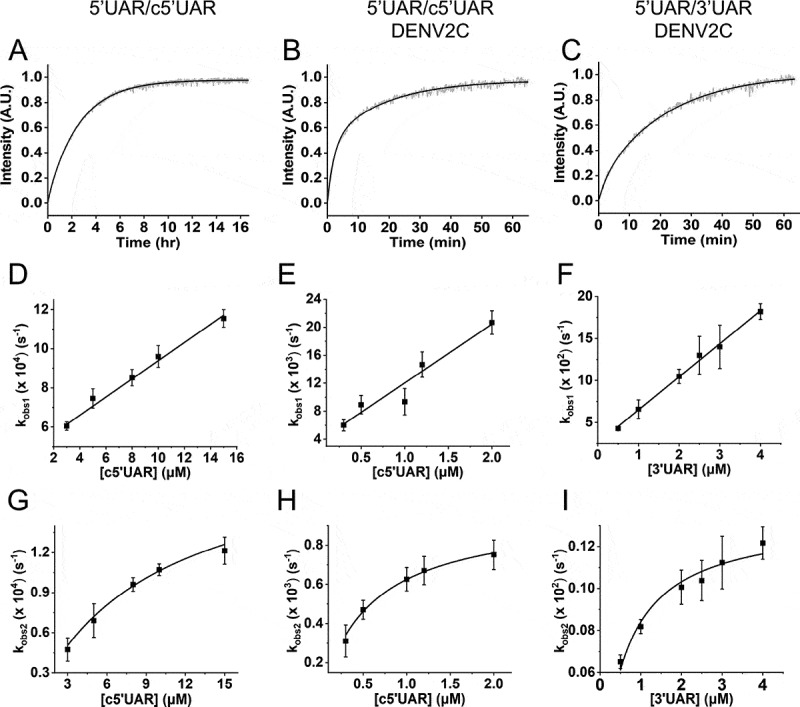
Real-time progress curves (A-C) and kinetic parameters (D-I) of annealing between 5ʹUAR and its complementary sequences (c5ʹUAR and 3ʹUAR) in the presence and absence of DENV2C. (A-C) Real-time progress curves (grey) and their fits to Equation (1) (black) to obtain fast (*k*_obs1_) and slow (*k*_obs2_) kinetic parameters. Real-time progress curve of the annealing between (A) 10 nM 5ʹUAR and 8 μM c5ʹUAR (B) 10 nM 5ʹUAR and 1 μM c5ʹUAR in the presence of DENV2C with DENV2C:ORN ratio of 2:1 and (C) 10 μM 5ʹUAR and 1 μM 3ʹUAR in the presence of DENV2C with DENV2C:ORN ratio of 2:1. The obtained values of *k*_obs1_ and *k*_obs2_ for (D and G) 5ʹUAR/c5ʹUAR, (E and H) 5ʹUAR/c5ʹUAR in presence of DENV2C and (F and I) 5ʹUAR/3ʹUAR in presence of DENV2C were plotted against corresponding complementary ORNs and fitted to Equation (2) and (3), respectively. Excitation and emission wavelengths used were 480 nm and 520 nm, respectively. Error bars show standard deviation from at least three repeats

**Figure 3.**
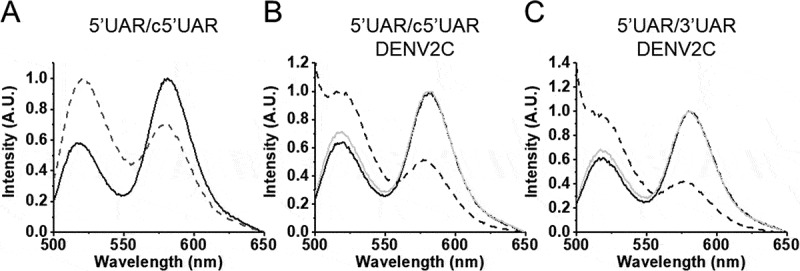
Emission spectra of 5ʹUAR before (solid black) and after annealing (dotted black) to its complementary sequences. (A) Emission spectra before and after annealing of 10 nM 5ʹUAR to 8 μM c5ʹUAR. (B) Emission spectra of 5ʹUAR in the absence (solid black) and presence (solid grey) of DENV2C in an DENV2C:ORN ratio of 2:1. The emission spectra after annealing of 10 nM 5ʹUAR to 1 μM c5ʹUAR in the presence of DENV2C is shown in dotted lines. (C) Emission spectra of 5ʹUAR in the absence (solid black) and presence (solid grey) of DENV2C in an DENV2C:ORN ratio of 2:1. The emission spectra after annealing of 10 nM 5ʹUAR to 3 μM 3ʹUAR in the presence of DENV2C is shown in dotted lines. The excitation wavelength was 480 nm

Annealing kinetics of doubly labelled 5ʹUAR to its non-labelled c5ʹUAR were monitored by tracking the increase in FAM fluorescence (Fig. 2A). Comparing the emission spectra of doubly labelled 5ʹUAR at the start and end of the annealing reaction shows a decrease in FRET with the formation of the extended duplex (Fig. 3A) as seen by an increase in FAM fluorescence and a corresponding decrease in TAMRA fluorescence. The real-time fluorescence intensity traces of the 5ʹUAR/c5ʹUAR reaction kinetics were fitted to a biexponential equation (1), where *I(t)* is the actual fluorescence intensity at 520 nm, upon excitation at 480 nm, *k*_obs1_ and *k*_obs2_ are the pseudo first order fast and slow reaction rates, *a* is the relative amplitude of the fast component and *t*_0_ is the start time of the reaction. *I_0_* and *I_f_* is the fluorescence intensity of doubly-labelled 5ʹUAR in the free state and in the final extended duplex (ED), respectively.

(1) $$I\left(t\right)=I_{f}-\left(I_{f}-I_{0}\right)\left(ae^{\left(-k_{obs1}\left(t-t0\right)\right)}+\left(1-a\right)e^{\left(-k_{obs2}\left(t-t0\right)\right)}\right)$$

The fast and slow reaction rates, *k*_obs1_ and *k*_obs2,_ were plotted against the concentration of non-labelled reactant, c5ʹUAR (Fig. 2D and 2G). The fast component, *k*_obs1_, linearly varies with increasing c5ʹUAR concentrations ([c5ʹUAR]), while the slow component, *k*_obs2_, shows a hyperbolic dependence. The linear relationship between *k*_obs1_ and [c5ʹUAR] follows Equation (2) [28].

(2) $$k_{obs1}=k_{ass}\left[c5\prime UAR\right]+k_{diss}$$

The hyperbolic dependence of *k*_obs2_ on increasing [c5ʹUAR] can be described in Equation (3), where K_a_ is the equilibrium constant governing intermediate complex (IC) formation, *k*_f_ and *k*_b_ are forward and backward interconversion kinetic rate constants.

(3) $$k_{obs2}=\frac{k_{f}K_{a}\left[c5\prime UAR\right]}{1+K_{a}\left[c5\prime UAR\right]}+k_{b}$$

Fitting the linear and hyperbolic plots of *k*_obs1_ and *k*_obs2_ against increasing [c5ʹUAR] with Equations (2) and (3), respectively, generated kinetic parameters shown in Table 1.

**Table 1.** **Kinetic parameters of 5ʹUAR/c5ʹUAR, 5ʹcHP/c5ʹcHP and 5ʹUAR/3ʹUAR annealing and their mutants in the absence and presence of DENV2C**. Kinetic rate constants were calculated from the dependence of *k*_obs_ values on the concentration of the unlabelled ORN. The *k*_ass_ and *k*_diss_ values were calculated with Equation (2), while the K_a_ and *k*_f_ values were calculated using Equation (3). The K_a_ values were found to differ by a factor of <1.5 from the *k*_ass_/*k*_diss_ values, which further supports the proposed reaction Scheme 1 and 2

Doubly labelled ORN	Complementary ORN	DENV2C:ORN ratio	*k*_ass_ (M^−1^s^−1^) × 10^−3^	*k*_diss_ (s^−1^) × 10^4^	K_a_ (M^−1^) × 10^−5^	*k*_f_ (s^−1^) × 10^4^
5ʹUAR	c5ʹUAR	0	0.05 (±0.003)	3.6 (± 0.7)	1.11 (± 0.19)	2.02 (± 0.17)
5ʹUAR	c5ʹUAR	2	8.4 (± 1.3)	35 (± 15)	16.8 (± 5.1)	9.6 (± 1.1)
5ʹUAR	3ʹUAR	2	39.4 (± 1.7)	255 (± 32)	16.9 (± 2.7)	13.4 (± 0.53)
5ʹcHP	c5ʹcHP	0	0.085 (±0.005)	2.6 (± 0.03)	2.3 (± 0.4)	0.5 (± 0.04)
5ʹcHP	c5ʹcHP	2	7.4 (± 0.8)	67 (± 9)	16 (± 4)	10.2 (± 1)

Based on our acquired kinetic parameters, a reaction mechanism with single kinetic pathway starting from a 5ʹUAR species can be proposed:

**Scheme 1:**
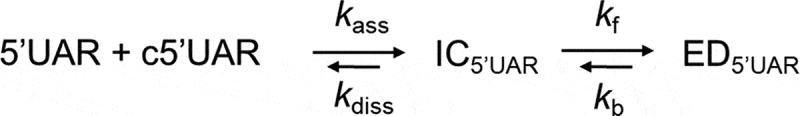

where a fast pre-equilibrium intermediate complex, IC_5ʹUAR_, precedes the formation of the final stable extended duplex, ED_5ʹUAR_, through a monomolecular reaction [29]. The formation of IC_5ʹUAR_ is governed by the second order association constant, *k*_ass_, and the first order dissociation constant, *k*_diss_, whereas the formation of ED_5ʹUAR_ is governed by the forward and backward interconversion kinetic rate constants, *k*_f_ and *k*_b_ respectively. The hyperbolic dependence of *k*_obs2_ with increasing [c5ʹUAR] can be attributed to IC_5ʹUAR_ accumulation because of its slow interconversion to ED_5ʹUAR_. This is likely the rate-limiting step of the 5ʹUAR/c5ʹUAR annealing.

The *k*_ass_ value of 50 (± 3) M^−1^s^−1^ for 5ʹUAR/c5ʹUAR annealing is at least 4 orders of magnitude smaller than the rate constants reported for annealing of unstructured sequences (10^5^–10^7^ M^−1^s^−1^), suggesting that there is a low probability for the reaction to occur at room temperature [30]. The *k*_diss_ and *k*_b_ values were 3.6 (± 0.7) × 10^−4^ s^−1^ and close to zero, respectively, suggesting that dissociation of both IC_5ʹUAR_ and ED_5ʹUAR_ is negligible (Table 1).

To validate the postulated annealing mechanism [31–34], we used the Dynafit numerical resolution software [35], which allows simultaneous fitting of the experimental progress curves obtained at different [c5ʹUAR] (Fig. S3). The best estimates of the elementary rate constants *k*_ass_, *k*_diss_ and *k*_f_ (Table S1) were in excellent agreement with those found by the empirical approach (Table 1).

Further insights into the nature of 5ʹUAR/c5ʹUAR annealing pathway were obtained from determining the temperature dependence of *k*_obs_ values, using the Arrhenius equation (4), which can be rewritten in a linear form [5], where the rate constant *k* is given by *k*_obs_/[c5ʹUAR], *A* is the pre-exponential Arrhenius factor, *E*_a_ is the activation energy, *R* is the universal gas constant and *T* is the temperature (in Kelvin).

(4) $$k=Ae^{-\frac{E_{a}}{RT}}$$

(5) $$\ln\frac{k_{obs}}{\left[c5^{\prime }UAR\right]}=\ln A-\frac{E_{a}}{R}\left(\frac{1}{T}\right)$$

**Figure 4.**
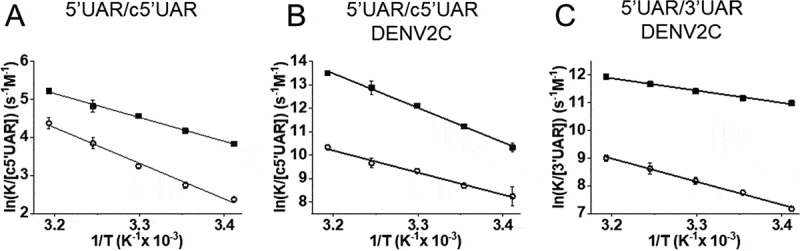
Temperature dependence of 5ʹUAR/c5ʹUAR and 5ʹUAR/3ʹUAR annealing in the (A) absence and (B and C) presence of DENV2C. The reactions between (A) 10 nM 5ʹUAR and 10 μM c5ʹUAR, (B) 10 nM 5ʹUAR and 500 nM c5ʹUAR in the presence of DENV2C and (C) 10 nM 5ʹUAR and 500 nM 3ʹUAR in the presence of DENV2C were monitored at various temperatures (20°C, 25°C, 30°C, 35°C and 40°C) to obtain fast, *k*_obs1_ and slow, *k*_obs2_ reaction rates. Natural logarithms of the fast (solid squares) and slow (open circles) reaction rates were plotted against the inverse of temperature. Solid lines are fits to Equation (5). The Ea obtained from fitting were used to calculate transition state enthalpies (Table 2) using $\Delta H=E_{a}-RT$with T = 293.15 K. Excitation and emission wavelengths were 480 nm and 520 nm, respectively. Error bars represents the standard deviations of at least three repeats

We observed an increase in both reaction rates leading to positive enthalpy values of the transition states of 11.9 (± 0.4) kcal/mol and 18.0 (± 1.0) kcal/mol for the fast and the slow components, respectively (Fig. 4 and Table 2). This suggests a pre-melting of 2–3 base-pairs (bp) of hydrogen bonds in 5ʹUAR hairpin structure [36,37].

**Table 2.** **Arrhenius parameters of 5ʹUAR/c5ʹUAR, 5ʹcHP/c5ʹcHP and 5ʹUAR/3ʹUAR annealing and their mutants in the absence and presence of DENV2C**. The values of the transition state enthalpies, ΔH for the fast and slow pathways were calculated from the fits of temperature dependence of reaction rates, *k*_obs_, values to Equation (5), as described in Fig. 4

Doubly labelled ORN	Complementary ORN	DENV2C:ORN ratio	ΔH (Fast) (kcal/mol)	ΔH (Slow) (kcal/mol)
5ʹUAR	c5ʹUAR	0	11.9 ± 0.4	18.0 ± 1.0
5ʹUAR	c5ʹUAR	2	28.6 ± 0.9	18.1 ± 1.1
5ʹUAR	3ʹUAR	2	8.2 ± 0.4	16.0 ± 0.6

Next, similar to 5ʹUAR/c5ʹUAR annealing, 5ʹcHP/c5ʹcHP annealing shows a linear and hyperbolic dependence of *k*_obs1_ and *k*_obs2_ values against increasing [c5ʹcHP] (Fig. S4), thus suggesting a similar reaction mechanism as to that proposed by Scheme 1 (shown by Scheme a in the supplementary). The formation of IC_cHP_ was found to be ~1.7-fold faster in 5ʹcHP/c5ʹcHP annealing compared to 5ʹUAR/c5ʹUAR annealing with *k*_ass_ values of 85 (±5) M^−1^s^−1^ and 50 (± 3) M^−1^s^−1^, respectively (Table 1). However, the interconversion from IC_cHP_ to ED_cHP_ is slower by ~4-fold for 5ʹcHP/c5ʹcHP annealing compared to that of 5ʹUAR/c5ʹUAR annealing (Table 1), which can be attributed to the greater stability of the 5ʹcHP hairpin, with a ΔG_cHP_ of −8.1 kcal/mol compared to a ΔG_5ʹUAR_ of −7.4 kcal/mol (Fig. 1).

As mentioned earlier, investigating 5ʹUAR/3ʹUAR annealing will provide insights about (+) RNA circularization. Interestingly, 5ʹUAR/3ʹUAR annealing was drastically slower as compared to both 5ʹUAR/c5ʹUAR and 5ʹcHP/c5ʹcHP annealing and no increase in donor emission was observed even after 12 hours (Fig. S1E). This indicates that the kinetic barrier during the extended duplex (ED_3ʹUAR_) formation for 5ʹUAR/3ʹUAR annealing is considerably higher as compared to both 5ʹUAR/c5ʹUAR and 5ʹcHP/c5ʹcHP annealing. This higher kinetic barrier also points towards the partial non-complementarity between 5ʹUAR and 3ʹUAR hairpins during 5ʹUAR/3ʹUAR annealing (Fig. S1B). Due to the extremely slow reaction kinetics of 5ʹUAR/3ʹUAR annealing, no kinetic parameters could be determined, at least under our experimental conditions (Fig. S1E).

### DENV2C accelerates all three 5ʹUAR/c5ʹUAR, 5ʹcHP/c5ʹcHP and 5ʹUAR/3ʹUAR annealing

To understand the RNA chaperone activity of DENV2C and its role in genome recombination, we characterized the annealing of 5ʹUAR/c5ʹUAR, 5ʹcHP/c5ʹcHP and 5ʹUAR/3ʹUAR in the presence of DENV2C. However, RNA chaperones are known to cause nucleic acid aggregation [31,32,38–40] which can result in fast kinetics that cannot be reliably measured (Fig. S5C) using fluorescence-based techniques [38,41]. Thus, we first determined non-aggregating experimental conditions. Nucleic acid aggregation by positively charged proteins is concentration dependent [30,42] and can be investigated using FCS [31,32]. In FCS, the fluorescence intensity arising from confocal volume (about 0.2 fL) is autocorrelated to obtain information about the processes that give rise to fluorescence fluctuations. These fluctuations are governed by the diffusion of fluorescent species in the confocal volume, and thus parameters like average number of fluorescent species and their diffusion constant can be determined. Aggregation of labelled 5ʹUAR molecules by the DENV2C would decrease the number of fluorescent oligoribonucleotides (ORNs), N and increase the diffusion time, τ_D_. By adding increasing concentrations of DENV2C to labelled ORN or labelled DENV2C, we found no change in N or τ_D_ up to a DENV2C:ORN molar ratio of 2:1, indicating that no aggregation occurred under these conditions (Fig. S5A and S5B). The optimized annealing reaction performed at a DENV2C:ORN molar ratio of 2:1 at 20°C allowed reliable and reproducible determination of kinetic parameters (further explanations about aggregation conditions are provided in Fig. S5). Thus, we selected a DENV2C:ORN molar ratio of 2:1 to characterize the annealing of 5ʹUAR/c5ʹUAR, 5ʹcHP/c5ʹcHP and 5ʹUAR/3ʹUAR in the presence of DENV2C.

Addition of DENV2C to the labelled 5ʹUAR or 5ʹcHP sequences did not lead to any significant change in their fluorescence spectrum (compare black and grey emission spectra in Fig. 3B,C), indicating that DENV2C was unable to destabilize the stem of their secondary structures, similar to other RNA chaperones [31,32]. Annealing of 5ʹUAR/c5ʹUAR, 5ʹcHP/c5ʹcHP and 5ʹUAR/3ʹUAR were then performed by adding DENV2C at a protein to ORN molar ratio of 2:1, to ensure aggregation-free conditions. Interestingly, DENV2C caused a dramatic increase in all the three annealing reactions, as both 5ʹUAR/c5ʹUAR and 5ʹcHP/c5ʹcHP annealing were completed much faster as compared to their respective annealing in the absence of the protein (Fig. S1D and S1F). Unlike in the absence of DENV2C, 5ʹUAR/3ʹUAR annealing was completed in ~1 hour in the presence of the protein (Fig. 2C and S3E). The kinetic traces could be adequately fitted with Equation (1) and kinetic parameters were obtained for DENV2C-promoted 5ʹUAR/c5ʹUAR, 5ʹcHP/c5ʹcHP and 5ʹUAR/3ʹUAR annealing (Table 1).

Similar to the absence of DENV2C, the fast component, *k*_obs1_, varied linearly and the slow component, *k*_obs2,_ showed hyperbolic dependence with increasing [c5ʹUAR] during DENV2C-promoted 5ʹUAR/c5ʹUAR annealing (Fig. 2E and 2H). This suggests a similar reaction mechanism as to that proposed by Scheme 1. Thus, similar to other RNA chaperone-promoted ORN annealing [32,43] and taking acquired kinetic data into account, a reaction mechanism with single kinetic pathway involving a single 5ʹUAR/DENV2C complex (5ʹUAR_1_) can be proposed:

**Scheme 2:**
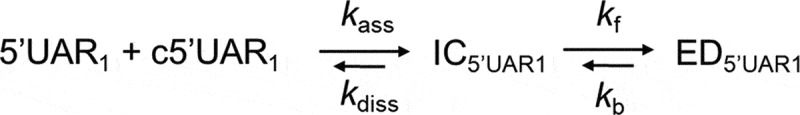

where a fast pre-equilibrium intermediate complex, IC_5ʹUAR1_, precedes the formation of the final stable extended duplex, ED_5ʹUAR1_, through a monomolecular reaction [29]. The formation of IC_5ʹUAR1_ is governed by the second order association constant, *k*_ass_, and the first order dissociation constant, *k*_diss_, whereas the formation of ED_5ʹUAR1_ is governed by the forward interconversion kinetic rate constant, *k*_f_. Again, we validated 5ʹUAR/c5ʹUAR annealing mechanism in the presence of DENV2C by using the Dynafit numerical resolution software (Table S1) [31–35].

To gain further insight into the annealing mechanism, we evaluated the temperature dependence of the *k*_obs_ values. The relationship revealed positive enthalpy values of the transition states of 28.6 (± 0.9) kcal/mol and 18.1 (± 1.1) kcal/mol for the fast and slow components, respectively (Fig. 4 and Table 2). These values indicate that 5ʹUAR/c5ʹUAR annealing promoted by the DENV2C involves pre-melting of ~5 to ~3 bp in the 5ʹUAR hairpin structure [36,37].

Next, the linear and the hyperbolic dependence of *k*_obs1_ and *k*_obs2_, respectively with increasing [c5ʹcHP] (Fig. S4F and S4H) during DENV2C-promoted 5ʹcHP/c5ʹcHP annealing implies that the reaction mechanism follows a single kinetic pathway scheme with an accumulation of IC_cHP1_ followed by its interconversion from ED_cHP1_, similar to the one proposed by scheme 1 (Scheme b in the supplementary). DENV2C-promoted 5ʹUAR/3ʹUAR annealing also showed linear and hyperbolic dependence of *k*_obs1_ and *k*_obs2_, respectively with increasing [3ʹUAR] (Fig. 2F,I), again indicating a reaction scheme similar to that of scheme 1 (Scheme c in the supplementary).

A comparison of the association constants, *k*_ass_, suggests DENV2C chaperones the rate of intermediate complex formation by ~2 orders of magnitude during both 5ʹUAR/c5ʹUAR and 5ʹcHP/c5ʹcHP annealing (Table 1). Similarly, in the presence of DENV2C, the rate of intermediate complex conversion to the extended duplex increases by ~5-folds for 5ʹUAR/c5ʹUAR annealing and by ~20-folds for 5ʹcHP/c5ʹcHP annealing (comparing corresponding *k*_f_ values in Table 1). Interestingly, we observed a ~ 5-fold increase in the *k*_ass_ values of the 5ʹUAR/3ʹUAR (39.4 × 10^3^ M^−1^s^−1^) annealing as compared to the *k*_ass_ value of the 5ʹUAR/c5ʹUAR (8.4 × 10^3^ M^−1^s^−1^) annealing in the presence of DENV2C. This indicates that the presence of DENV2C probably lowers the kinetic barrier, present due to the non-complementary intermolecular base pairs between 5ʹUAR and 3ʹUAR hairpins (Fig. S1B). This result is also in line with the lower enthalpy values obtained for the transition states of 8.2 (± 0.4) kcal/mol for the fast component (Fig. 4 and Table 2) during DENV2C-promoted 5ʹUAR/3ʹUAR annealing and implies that the formation of IC_3ʹUAR1_ requires the pre-melting of only ~2 bp (Scheme c in the supplementary) as compared to the ~5 bp during the formation of IC_5ʹUAR1_ (Table 1 and Scheme 2). Although IC_3ʹUAR1_ formed rapidly as compared to IC_5ʹUAR1_, the ~8-folds increase in its dissociation rate constant, *k*_diss_ showed that the structural stability of such intermediate complex is substantially low, probably due to the intermolecular base pair mismatches in the 5ʹUAR/3ʹUAR duplex as compared to the 5ʹUAR’c5ʹUAR duplexes (Fig. S1A and S1B).

### DENV2C switches nucleation of 5ʹUAR/c5ʹUAR and 5ʹcHP/c5ʹcHP annealing through kissing-loop intermediates to stem-stem interactions but not for 5ʹUAR/3ʹUAR annealing

**Figure 5.**
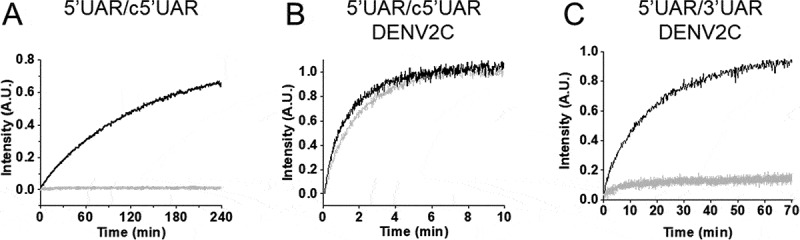
Real-time progress curves of the 5ʹUAR mutants. Progress curve of dual-labelled 5ʹUAR (black) or 5ʹUAR-ULoop mutant (grey) with its complementary sequences (A and B) c5ʹUAR or (C) 3ʹUAR in the absence and presence of DENV2C, respectively. (A) 10 μM c5ʹUAR, (B) 700 nM c5ʹUAR and (C) 1 μM 3ʹUAR were used for the annealing reaction with either 10 nM of 5ʹUAR (black traces) or 10 nM of 5ʹUAR-ULoop (grey traces). (B) Fitting of the progressive curves using Equation (1) provided values for the 5ʹUAR/c5ʹUAR reaction in the absence (*k*_obs1_ and *k*_obs2_ are 4.0 × 10^−2^ s^−1^ and 4.4 × 10^−3^ s^−1^) and the presence (*k*_obs1_ and *k*_obs2_ of 4.5 × 10^−2^ s^−1^ and 4.7 × 10^−3^ s^−1^) of DENV2C. Excitation and emission wavelengths used were 480 nm and 520 nm, respectively

We characterize the molecular mechanisms of 5ʹUAR/c5ʹUAR and 5ʹcHP/c5ʹcHP annealing by investigating the role of the hairpin loop. To determine whether annealing was nucleated through kissing-loop intermediates, we used 5ʹUAR-ULoop and 5ʹcHP-ULoop mutants, where the G_9_, C_10_, A_11_, G_12_, A_13_ and A_10, 11, 12 and 13_, C_14_ residues, respectively, were changed to U residues (Fig. 1B) in order to decrease the complementarities between the central loops. For both 5ʹUAR-ULoop/c5ʹUAR and 5ʹcHP-ULoop/c5ʹcHP annealing reactions, no increase in FAM fluorescence was observed (Fig. 5A and Fig. S6A). This drastic decrease in annealing reactions with the loop mutants strongly suggest that both 5ʹUAR/c5ʹUAR and 5ʹcHP/c5ʹcHP annealing reactions are mainly nucleated through the kissing-loop intermediates. In contrast, in the presence of DENV2C both 5ʹUAR-ULoop/c5ʹUAR and 5ʹcHP-ULoop/c5ʹcHP kinetic reactions were found to be similar to that of DENV2C-promoted 5ʹUAR/c5ʹUAR and DENV2C-promoted 5ʹcHP/c5ʹcHP annealing (Fig. 5B and Fig. S6B), respectively. No decrease in annealing reaction rates (values provided in Fig. 5 legend) with the loop mutants indicate that the role of hairpin loops in DENV2C-promoted 5ʹUAR/c5ʹUAR and DENV2C-promoted 5ʹcHP/c5ʹcHP annealing is limited and hence, both kinetic reactions are prominently nucleated through the stems. Interestingly, it was observed that even in aggregation conditions of DENV2C:ORN of 10:1, the annealing mechanism of 5ʹUAR/c5ʹUAR and 5ʹUAR/3ʹUAR remained predominantly via stem-stem and kissing-loop interactions respectively (Fig. S6C and S6D).

Taken together, these results showed that DENV2C switches the annealing mechanism of RNA elements, 5ʹUAR and 5ʹcHP, during (+)/(-) ds-RNA formation from predominantly through kissing-loop intermediates to stem-stem interactions. It is possible that hydrogen bond melting in the stem region of the 5ʹUAR or c5ʹcHP hairpin increases due to its interaction with DENV2C protein, and this leads to the switching of the annealing mechanism. Interestingly, a drastic decrease in DENV2C-promoted 5ʹUAR-ULoop/3ʹUAR annealing was observed as compared to its counterpart, DENV2C-promoted 5ʹUAR/3ʹUAR annealing (Fig. 5C). The result indicates a vital role of the hairpin loop in DENV2C-promoted 5ʹUAR/3ʹUAR annealing. Taken together, the result states that although DENV2 accelerates 5ʹUAR/3ʹUAR annealing, the role of the hairpin loop remains crucial during annealing. Thus, the annealing of 5ʹUAR with its complementary sequence during (+) RNA circularization, 3ʹUAR, predominantly propagates through loop intermediates. Overall, the results suggest that DENV2C altered the role of hairpin loops in annealing pathways of 5ʹUAR/c5ʹUAR and 5ʹcHP/c5ʹcHP as compared to their annealing in absence of the protein but not for the 5ʹUAR/3ʹUAR annealing. Whilst beyond the scope of the present study, it would be of interesting to quantify the role of hairpin stems in the annealing pathways.

### DENV2C protein facilitates 5ʹUAR/c5ʹUAR and 5ʹUAR/3ʹUAR annealing by decreasing the intrinsic dynamics of the 5ʹUAR hairpin

**Figure 6.**
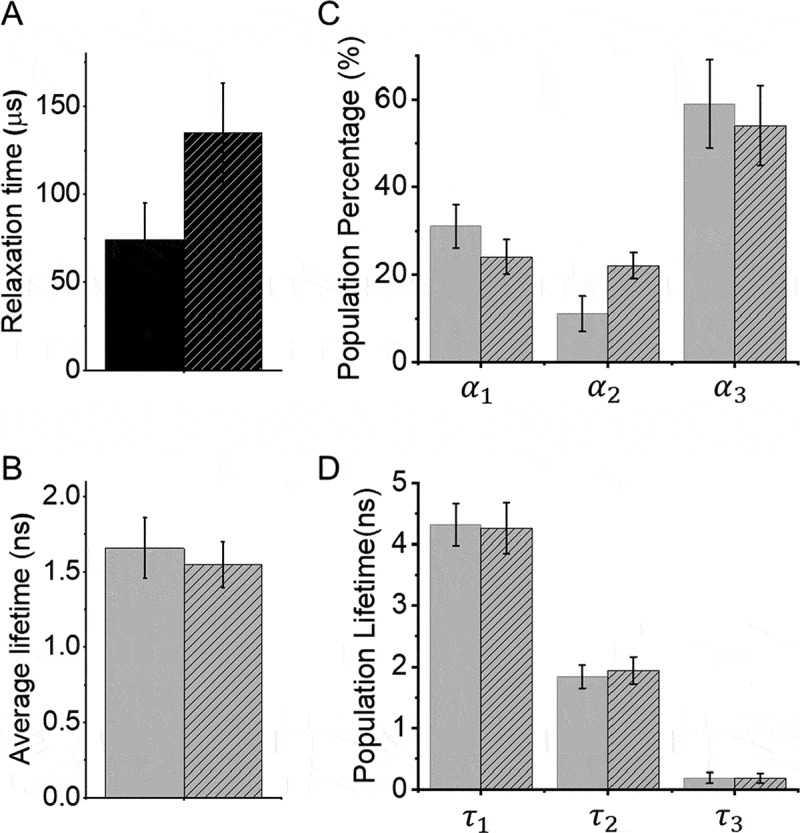
FRET-FCS and trFRET of 5ʹUAR in the presence and the absence of DENV2C

To further understand how DENV2C chaperones 5ʹUAR/c5ʹUAR and 5ʹUAR/3ʹUAR annealing during (+)/(-) ds-RNA formation and (+) RNA circularization respectively, we measured the intrinsic dynamics of 5ʹUAR in the presence and absence of the protein. We used FRET-FCS [44,45] which analyses the fluctuations in FRET efficiency, caused by doubly labelled 5ʹUAR conformational fluctuations. Unlike FCS, FRET-FCS measurers the proximity ratio (*p*), which is a function related to FRET efficiency [45–50] and depends on the separation between donor and acceptor but not on the position of the molecules in the observation volume. Thus, the correlation function of the proximity ratio *p* (*Gp*) provides information about structural dynamics of the various 5ʹUAR conformations. In this case, the 5ʹUAR might exist in closed, partially-open and fully-open hairpin conformations and thus, the correlation function of *p* can be fitted to a stretched exponential equation (Fig. 6). The equation provides an effective relaxation time (τ_p_), and a stretch parameter (β) describing the heterogeneity of the system. A value of 1 for β indicates that the system displays normal two-state Arrhenius kinetics, with one discrete energy barrier, while a value of 0 for β indicates a continuum of equal energy barriers. The approach was calibrated as mentioned by Sharma KK et al. [49] and effective relaxation time (τ_p_) associated with the motion of the 5ʹUAR hairpin extremities, were determined. Addition of DENV2C to the 5ʹUAR hairpin resulted in a ~ 2-fold increase in τ_p_ (from 74 ± 21 μs in the absence to 135 ± 28 μs in the presence of DENV2C) (Fig. 6A), suggesting the hampered fluctuation of 5ʹUAR hairpin extremities. The β values of 0.21 (± 0.04) in the absence and 0.25 (± 0.06) in the presence of DENV2C showed that the 5ʹUAR hairpin exists in more than 2 conformations and that the addition of DENV2C does not alter heterogeneity of the system. However, it is possible that one of the 5ʹUAR hairpin conformations is favoured in the presence of the protein.

(A) The relaxation time, τ*_p_*, of 5ʹUAR in the absence (solid) and presence (striped) of DENV2C. An autocorrelation function of proximity ratio, *G_P_(τ)*, was constructed using: $G_{P}\left(\tau_{p}\right)=\frac{\delta P\left(t\right)\delta P\left(t+\tau_{p}\right)}{{\left\langle P\left(t\right)\right\rangle }^{2}}$ and fitted to a stretched exponential equation [47]: $G_{P}\left(\tau_{p}\right)=G_{P}\left(0\right)exp\left[-{\left(\frac{t}{\tau_{p}}\right)}^{\beta }\right]$,where τ*_p_* corresponds to the effective relaxation time associated with the correlated motion and β is a stretch parameter. (B) The lifetime traces for doubly labelled 5ʹUAR were fitted to a tri-exponential decay model and the average fluorescence lifetimes (<τ_f_>) of FAM for donor were calculated in the (solid) absence and in the (striped) presence of DENV2C. (C and D) Fitting of the fluorescence decay provided three discrete lifetimes, τ_1_, τ_2_ and τ_3_ with the corresponding fraction of each population as α_1_, α_2_ and α_3_, respectively in the (solid) absence and (striped) the presence of DENV2C added at protein:ORN ratio of 2:1. Error bars represents standard deviations of at least three repeats.

We investigated the notion of the favoured 5ʹUAR conformation among its various conformations using trFRET. In trFRET, the energy transfer from donor to acceptor influences the donor fluorescence lifetime, τ, in a distance-dependent manner. The closer the donor and acceptor dyes are, the faster the donor dye relaxes to ground state and the shorter the lifetime. The fluorescence of 5ʹUAR had an average lifetime of 1.7 ± 0.2 ns (Fig. 6B) and was best fitted with three discrete lifetime components of ~4.3 ns (<τ_1_>), ~1.9 ns (<τ_2_>) and ~0.19 ns (<τ_3_>) having populations of 31 ± 5% (α_1_), 11 ± 4% (α_2_) and 59 ± 10% (α_3_), respectively (Fig. 6C and 6D). Note that we cannot exclude the existence of conformations with very short lifetimes or with lifetimes close to the three principal components. Those would not be resolvable within our experimental conditions. We refer to the fluorescence lifetime of closed, partially-open and fully-open conformations of the 5ʹUAR hairpins as <τ_3_>, <τ_2_> and <τ_1_> and their corresponding populations as α_3_, α_2_ and α_1_.

No or marginal difference in the average lifetime of the donor was observed after addition of DENV2C to the 5ʹUAR hairpin. However, a ~ 2-fold increase in the value of α_2_ from 11 ± 4% to 22 ± 3% was observed that happened at the expense of both α_1_ and α_3_ populations (Fig. 6C). The α_1_ and α_3_ populations showed a decrease from 31 ± 5% to 24 ± 4% and from 59 ± 10% to 54 ± 9% respectively. The result indicates that as an RNA chaperone with the ability to both dissociate and anneal RNA [20], DENV2C could be effectively unwinding high FRET populations (<τ_1_> and α_1_) and annealing low FRET populations (<τ_3_> and α_3_) to allow the 5ʹUAR hairpin to reach its most favoured kinetic conformation (<τ_2_> and α_2_). Overall, the results from FRET-FCS and trFRET explain that DENV2C probably exerts its RNA chaperone activities on the 5ʹUAR by modulating its intrinsic dynamics as well as by decreasing kinetically trapped unfavourable conformations. Such mechanistic behaviour of the DENV2C is in line with the ‘entropy exchange model’ in which a highly flexible protein, like DENV2C, undergoes disordered-to-ordered transition upon binding to RNA, that in turn leads to the melting of the RNA structure through an entropy exchange process [23].

## Discussion

**Figure 7.**
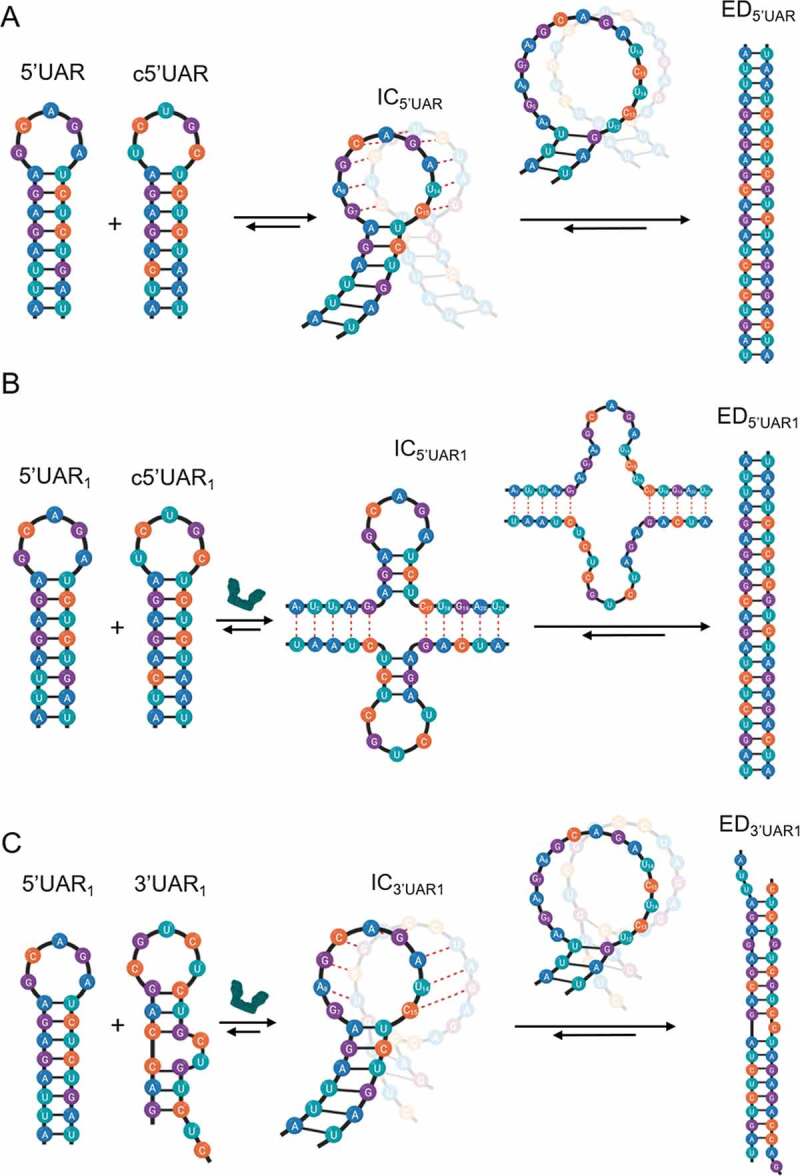
Proposed reaction mechanisms for annealing of 5ʹUAR/c5ʹUAR and 5ʹUAR/3ʹUAR in presence and absence of DENV2C. (A) Elucidating the proposed mechanism for 5ʹUAR/c5ʹUAR annealing involves first, the annealing kinetics showing an accumulation of an intermediate complex (IC_5ʹUAR_) and thus, suggesting a one-pathway reaction scheme. Annealing is nucleated via a kissing-loop intermediate (IC_5ʹUAR_), consisting of hydrogen bonding between complementary nucleotides in the loops of the 5ʹUAR and the c5ʹUAR (red dashed line). Formation of IC_5ʹUAR_ involves melting of two base pairs (G_7_-C_15_ and A_8_-U_14_) near the bottom of the 5ʹUAR loop. After which, interconversion from IC_5ʹUAR_ to ED_5ʹUAR_ takes place, likely involving the melting of the next three base pairs (A_6_-U_16_, G_5_-C_17_ and A_4_-U_18_) in the middle region of the 5ʹUAR stem. (B) DENV2C-promoted 5ʹUAR/c5ʹUAR annealing starts with the opening of the 5ʹUAR hairpin in the stem region, allowing annealing via the stems. Formation of IC_5ʹUAR1_ involves melting of the five base pairs (A_1_-U_21_, U_2_-A_20_, U_3_-G_19_, A_4_-U_18_ and G_5_-C_17_) in the lower 5ʹUAR stem. Interconversion from IC_5ʹUAR1_ to ED_5ʹUAR1_ involves melting of the three remaining base pairs (A_6_-U_16_, G_7_-C_15_ and A_8_-U_14_) in the upper part of 5ʹUAR stem. (C) DENV2C-promoted 5ʹUAR/3ʹUAR annealing takes place predominantly through the kissing-loop intermediates, with IC_3ʹUAR1_ formation likely involving the melting of the two base pairs (G_7_-C_15_ and A_8_-U_14_) near the bottom of the 5ʹUAR loop. Interconversion from IC to ED likely involves the melting of the three remaining base pairs (A_6_-U_16_, G_5_-C_17_ and A_4_-U_18_) in the middle region of the 5ʹUAR stem

The annealing reaction of the essential RNA element, 5ʹUAR, propagates through kissing-loop intermediates, albeit rather slowly, taking several hours to reach completion (Fig. 2A and Fig. S1A). This slow annealing kinetics can be related to the requirement of complementary sequences to be in a reactive conformation and proper orientation in order to nucleate the intermediate complex. The melting of two base pairs near the bottom of the 5ʹUAR are associated with IC_5ʹUAR_ formation. The IC_5ʹUAR_ is then converted into the ED_5ʹUAR_ in a rate limiting step associated with the transition enthalpy of ~18 kcal mol^−1^, that likely corresponds to the melting of the three next base pairs in the middle region of the 5ʹUAR stem. This melting is probably the bottleneck for the interconversion into ED_5ʹUAR_ (Fig. 7A).

Interestingly, in the presence of DENV2C, the annealing mechanism of 5ʹUAR/c5ʹUAR switched from mainly nucleating through kissing-loop intermediates and proceeded via stem-stem interactions. Formation of 5ʹUAR/c5ʹUAR intermediates in the presence of DENV2C probably involves melting of the five base pairs in the lower 5ʹUAR stem and the intermediate complex (IC_5ʹUAR1_) could be stabilized by up to 10 intermolecular base pairs (Fig. 7B). Moreover, the ~5-fold increased interconversion rate of IC_5ʹUAR1_ probably results from its stability due to the larger number of intermolecular base pairs and/or more favourable conformation for the subsequent conversion into the ED_5ʹUAR1_.

In contrast, the vital role of the loop in DENV2C-promoted 5ʹUAR/3ʹUAR annealing forced the reaction mechanism to proceed through the kissing-loop intermediate, IC_3ʹUAR1_. Thus, similar to the 5ʹUAR/c5ʹUAR intermediates in the absence of the protein, the formation of 5ʹUAR/3ʹUAR intermediates involves melting two base pairs in the upper region of the 5ʹUAR stem and the resulting intermediate complex could be stabilized by up to eight intermolecular base pairs followed by its interconversion to the extended duplex by the melting of the next three base pairs in the middle region of the 5ʹUAR stem (Fig. 7C). The initial three bp at the 5ʹ end of the 5ʹUAR stem are non-complementary to the corresponding nucleotides in the 3ʹUAR stems (Fig. S1B). This may hinder the propagation of DENV2C-promoted 5ʹUAR/3ʹUAR annealing through the hairpin stems. In addition, the lower transition enthalpy of ~8 kcal mol^−1^ (Table 2) indicated a rapid formation of IC_3ʹUAR1_ and in turn a ~ 5-fold increased association constant compared to DENV2C-promoted 5ʹUAR/c5ʹUAR annealing (Table 1). However, IC_3ʹUAR1_ is probably stabilized with only ~five intermolecular base pairs (involving A_8_, G_9_, C_10_, A_11_ and G_12_ of 5ʹUAR hairpin) which lead to its ~8-fold increased dissociation rate constant compared to the DENV2 promoted 5ʹUAR/c5ʹUAR annealing (Table 1). Apart from reduced intermolecular base pairing, the inferior stability of IC_3ʹUAR1_ could also be attributed to non-complementarities at the bottom of the 5ʹUAR loop involving G_7 and 12_ (Fig. S1B) and melting of three base pairs in the stable 5ʹUAR stem. Therefore, the combination of mismatches defining the stability of the ORN duplexes and the structured RNA elements chaperoned by DENV2C during ORN annealing (either hairpin loop or hairpin stem) probably dictates the processes of annealing of RNA elements representing (+)/(-) ds-RNA formation and (+) RNA circularization.

Our trFRET data showed that DENV2C functions similarly to other RNA chaperones [19,51–56] by binding to RNA, allowing them to escape thermodynamically stable, but possibly non-functional states and thus, explore other states that could be functional [57]. A comparison between different RNA chaperones suggests that DENV2C, HIV-1 NCp7 and hepatitis C virus (HCV) core protein accelerates annealing of complementary ORNs up to 2 to 3 orders of magnitude [31,32,58], indicating similar chaperoning potential for the three proteins. However, DENV2C accelerates annealing of complementary ORNs at two equivalent of protein molecule while HIV-1 NCp7 and HCV core require ~six and ~two equivalent of protein molecules, respectively, to show similar chaperone potential. This suggests that one molecule of either DENV2C or HCV core could be as active as three molecules of HIV-1 NCp7. Interestingly, both DENV2C and HCV core are existing as homodimers while HIV-1 NCp7 is a monomeric protein *in vitro* [59,60]. Thus, like HCV core, the superior chaperone activity of DENV2C compared to HIV-1 NCp7 is likely a consequence of the stronger ‘nucleic acid aggregating’ properties, in line with the efficient oligonucleotide aggregation observed by FCS (Fig. S5). This propensity of the DENV2C to neutralize the negatively charged nucleic acids and to promote their aggregation is probably related to the highly flexible and unstructured helix 1 [22,59,61] as compared to the folded HIV-1 NCp7 structure with two zinc fingers [60,62]. Moreover, HIV-1 NCp7 promotes ORN annealing through kissing loop intermediates [29] while the HCV core mainly chaperone the annealing through the stems [31], the DENV2C can propagate annealing either through kissing-loop intermediates (for 5ʹUAR/3ʹUAR) or through the stems (for 5ʹUAR/c5ʹUAR and 5ʹcHP/c5ʹcHP). Taken together, our results show that the DENV2C has stronger nucleic acid annealing activity at low protein:ORN ratios when compared to HIV-1 NCp7, and it can modulate annealing pathways when compared to HCV core. Despite the structural differences between these three proteins, the conserved nucleic acid chaperone properties suggest that they are required in different viruses, in line with the conservation of RNA chaperoning in *Flaviviridae* core proteins [21].

Such properties are not observed in other DENV proteins. Other viral proteins which are essential for RNA replication are NS5 polymerase and NS3 helicase. However, the ribozyme activity assay showed that both NS5 polymerase and NS3 helicase have no or limited RNA chaperoning activity [20]. Another viral protein, NS2A is also shown to interact with RNA. However, NS2A is proposed to be involved in virion assembly, by recruiting the genome via binding to 3ʹUTR [63]. Since these tested viral RNA-interacting proteins, including DENV2C, are targeting the same RNA in carrying out their functions, it is conceivable that the RNA affinity of these proteins is regulated in maintaining the balance between different events in the viral life cycle. However, DENV2C is likely to be the only viral protein responsible for preventing misfolding of RNA during replication. In addition, there is a possibility of DENV2C playing a role in modulating (+)/(-) ds-RNA formation and (+) RNA circularization by controlling the annealing mechanisms of essential RNA elements. These abilities of DENV2C may be critical for genomic RNA dimerization in DENV replication and RNA packaging as well as in facilitating recombination between various DENV genotypes and subtypes to increase viral variability.

## Supplementary Material

Supplemental Material
