# Supplementary material for: Dengue virus strain 2 capsid protein switches the annealing pathway and reduces intrinsic dynamics of the conserved 5’ untranslated region
Source: RNA Biol. 2021 Jan 7;18(5):718–31. doi: 10.1080/15476286.2020.1860581 (PMC8078513; doi:10.1080/15476286.2020.1860581)
Supplement: Supplemental Material [file KRNB_A_1860581_SM4269.docx]

**Dengue virus strain 2 capsid protein switches the annealing pathway and reduces intrinsic dynamics of the conserved 5' untranslated region**

Xin Ee Yong^1,2^, Palur Venkata Raghuvamsi^3^, Ganesh S. Anand^3^, Thorsten Wohland^2,3*^, Kamal K. Sharma^2,3*^

^1^NUS Graduate School for Integrated Sciences and Engineering, National University of Singapore, 21 Lower Kent Ridge Road, Singapore 119077, Singapore.

^2^Centre for Bioimaging Sciences, National University of Singapore, 14 Science Drive 4, Singapore 117557, Singapore.

^3^Department of Biological Sciences, National University of Singapore, 14 Science Drive 4, Singapore 117543, Singapore.

*To whom correspondence should be addressed. Kamal K. Sharma, Tel: +65 82906620; Email: [dbskks@nus.edu.sg](mailto:dbskks@nus.edu.sg). Correspondence may also be addressed to Thorsten Wohland. Tel: +65 65161248; Fax: +65 67767882; Email: twohland@nus.edu.sg.

**Figure S1 – Extended duplex structures and comparison of annealing kinetics in the absence and presence of DENV2C.** Extended duplexes (ED) of (A) 5’UAR/c5’UAR, (B) 5’UAR/3’UAR and (C) 5’cHP/c5’cHP. A dash indicates complementary base pairing. The strand on the left is the doubly labeled strand: 5’UAR or 5’cHP. Below each duplex is the Gibbs free energy, ΔG associated with the hybridization of the duplex in kcal/mol. This is determined using the Two-state melting (hybridization) webtool from http://unafold.rna.albany.edu/. Graphs show the comparison between (D) 5’UAR/c5’UAR, (E) 5’UAR/3’UAR and (F) 5’cHP/c5’cHP reactions in the absence (light grey) and presence (black) of DENV2C. (D) Intensity traces of annealing between 10 nM 5’UAR and 8 μM c5’UAR (light grey), and 1 μM c5’UAR in the presence of DENV2C (black). (E) Intensity traces of annealing between 10 nM 5’UAR and 5 μM 3’UAR (light grey), and 3 μM 3’UAR in the presence of DENV2C (black). (F) Intensity traces of annealing between 10 nM 5’cHP and 10 μM c5’cHP (light grey), and 1 μM c5’cHP in the presence of DENV2C (black).

**
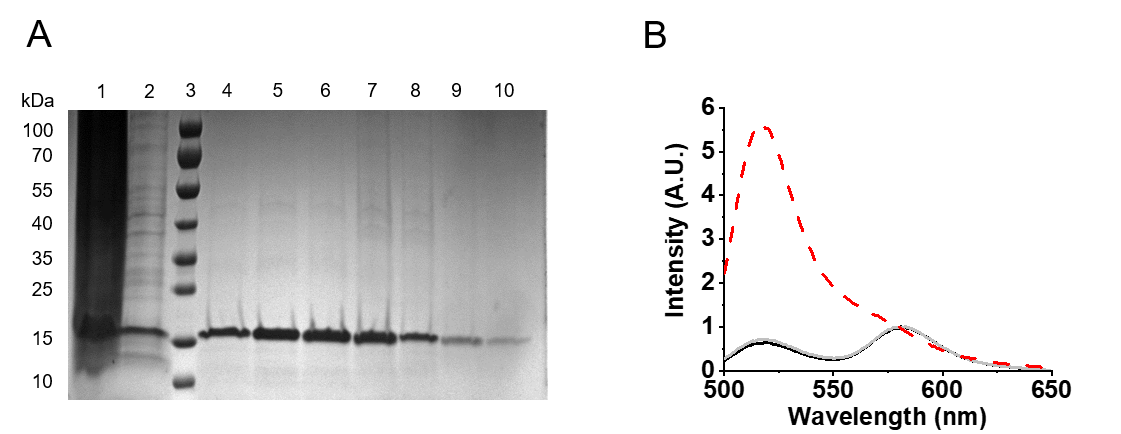
**

**Figure S2 – DENV2C protein is pure and ribonuclease-free.** (A) SDS-PAGE gel of affinity chromatography-purified DENV2C. Lanes show flowthrough (Lane 1), wash (Lane 2), DNA ladder (Lane 3) and elutions (Lanes 4–10). Elutions show a single band slightly above the 15 KDa PAGE gel DNA marker. The molecular weight corresponding to each band of the ladder is shown on the left of the gel image. (B) Emission spectra of 5’UAR (solid black) in the presence of DENV2C in an DENV2C:ORN ratio of 2:1 (solid grey), and in the presence of 100 ng/mL RNase A (dashed red). Spectra with DENV2C and RNase A were taken 5 minutes after incubation with 5’UAR at 20^o^C. The excitation wavelength was 480 nm. RNase A cleaves 5’UAR, separating the donor and acceptor dyes. Negligible changes to the emission spectra of 5’UAR in presence of DENV2C as compared to that in the presence of RNase prove that the protein is devoid of any RNase contamination.

**Dynafit simulation procedure**

Simulated progress curves were obtained at different concentration of c5’UAR (1 µM, 3 µM, 5 µM, 8 µM, 10 µM, 15 µM, 20 µM, 25 µM, 30 µM, 40 µM and 50 µM) using elementary rate constants, *k*_obs1_, *k*_obs2_ and *k*_f_, from Table 1 and according to the following Scheme [1–3]:


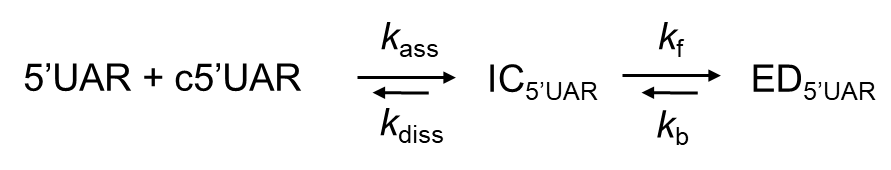


To obtain simulated progress curves, the concentration of 5’UAR was kept at a constant value of 10 nM. Progress curves of the 5’UAR/c5’UAR reaction were fitted to Equation (1) to obtain values for *k*_obs1_ and *k*_obs2_ with increasing concentration of c5’UAR. The fast and slow reaction rates, *k*_obs1_ and *k*_obs2,_ were plotted against the concentration of c5’UAR (Fig. S1A and Fig. S1B) and fitted using Equation (2) and (3), respectively. The generated kinetic parameters are shown in Table S1.


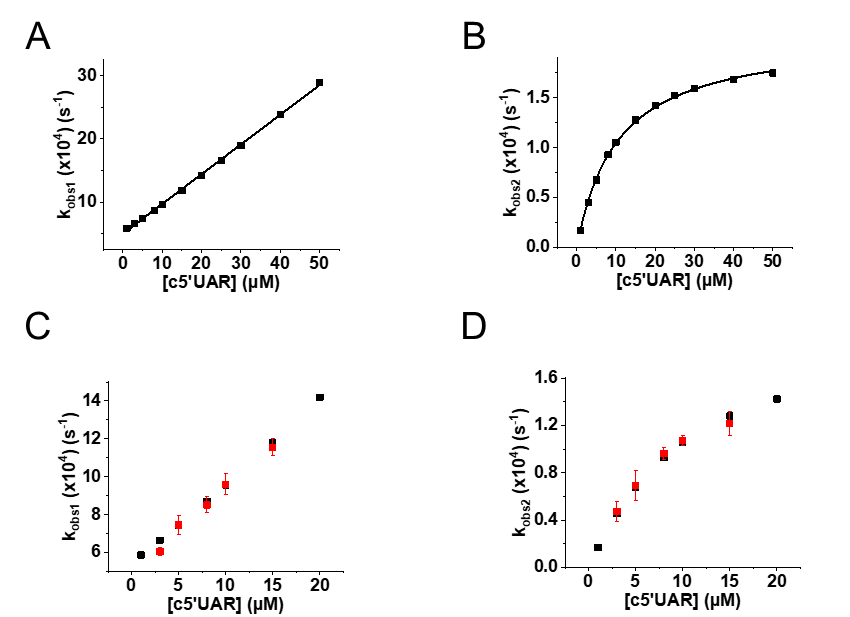


**Figure S3 – Simulated kinetic parameters of 5’UAR/c5’UAR annealing.** The fast (*k*_obs1_) (A) and slow (*k*_obs2_) (B) components for 5’UAR/c5’UAR annealing were determined by fitting simulated progress traces, which were obtained by using empirical kinetic parameters in Table 1 and increasing concentrations of c5’UAR. The solid lines correspond to the fit of the fast component (A) with Equation (2) and slow component (B) with Equation (3). The values are provided in Table S1. Comparison between kinetic parameters obtained for fast (C) and slow (D) components using experimental (red squares) and simulated (black squares) approach. The similar values of simulated kinetic parameters to that of experimental kinetic parameter values support the proposed reaction Schemes in the article.

**Table S1 – Kinetic parameters of 5’UAR/c5’UAR, 5’cHP/c5’cHP and 5’UAR/3’UAR annealing and their mutants in the absence and presence of DENV2C obtained by fitting simulated progression curves.**

| Doubly labeled ORN | Complementary ORN | DENV2C:ORN ratio | *k*_ass_  (M^-1^s^-1^) × 10^-3^ | *k*_diss_  (s^-1^) × 10^4^ | K_M_  (M^-1^) × 10^-5^ | *k*_f_  (s^-1^) × 10^4^ |
| --- | --- | --- | --- | --- | --- | --- |
| 5’UAR | c5’UAR | 0 | 0.047 (±0.005) | 5 (± 0.1) | 0.96 (± 0.03) | 2.1 (± 0.02) |
| 5’UAR | c5’UAR | 2 | 8.3 (± 0.02) | 39 (± 0.05) | 20.4 (± 0.4) | 9.7 (± 0.03) |
| 5’UAR | 3’UAR | 2 | 39.2 (± 0.02) | 260 (± 0.5) | 14.5 (± 0.1) | 13.6 (± 0.03) |
| 5’cHP | c5’cHP | 0 | 0.084 (±0.001) | 2.8 (± 0.03) | 2.9 (± 0.03) | 0.51 (± 0.01) |
| 5’cHP | c5’cHP | 2 | 7.3 (± 0.01) | 73 (± 0.05) | 9.8 (± 0.1) | 10.6 (± 0.03) |

Kinetic rate constants were calculated from the dependence of the *k*_obs_ values on the concentration of the unlabeled complementary ORN, as indicated in Figure S1. The *k*_ass_ and *k*_diss_ values were calculated using Equation (2), while the K_M_ and *k*_f_ values were calculated using Equation (3). Simulated kinetic parameters and empirical values (Table 1) were similar, supporting the proposed reaction Schemes in the article.

**Supplementary Schemes**


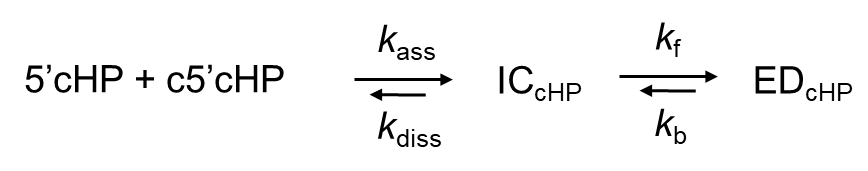
 **Scheme a**


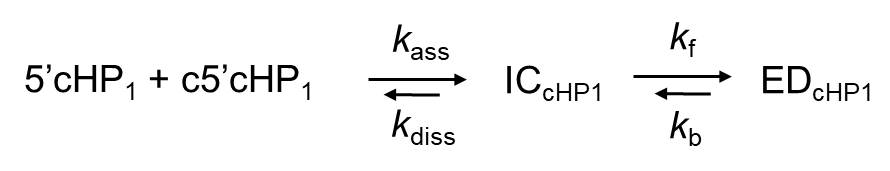
 **Scheme b**


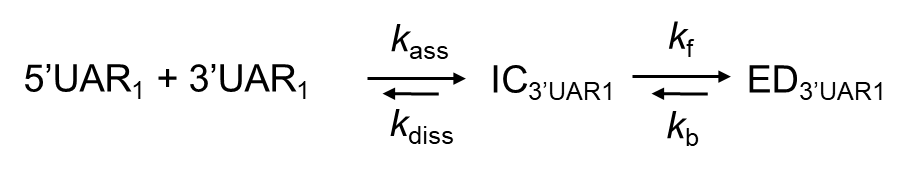
 **Scheme c**

According to the above-mentioned reaction schemes, a reaction mechanism with a single kinetic pathway involving a single initiation complex (ORN alone, 5’cHP, or in complex with DENV2C, 5’cHP_1_ and 5’UAR_1_) is proposed. In the reactions, a fast pre-equilibrium intermediate complex, IC_cHP_, IC_cHP1_ and IC_3’UAR1_, precedes the formation of the final stable extended duplexes, ED_cHP_, ED_cHP1_ and ED_3’UAR1_, through a monomolecular reaction. The formation of ICs are governed by the second order association constants, *k*_ass_, and the first order dissociation constants, *k*_diss_, whereas the formation of EDs are governed by the forward interconversion kinetic rate constants, *k*_f_.


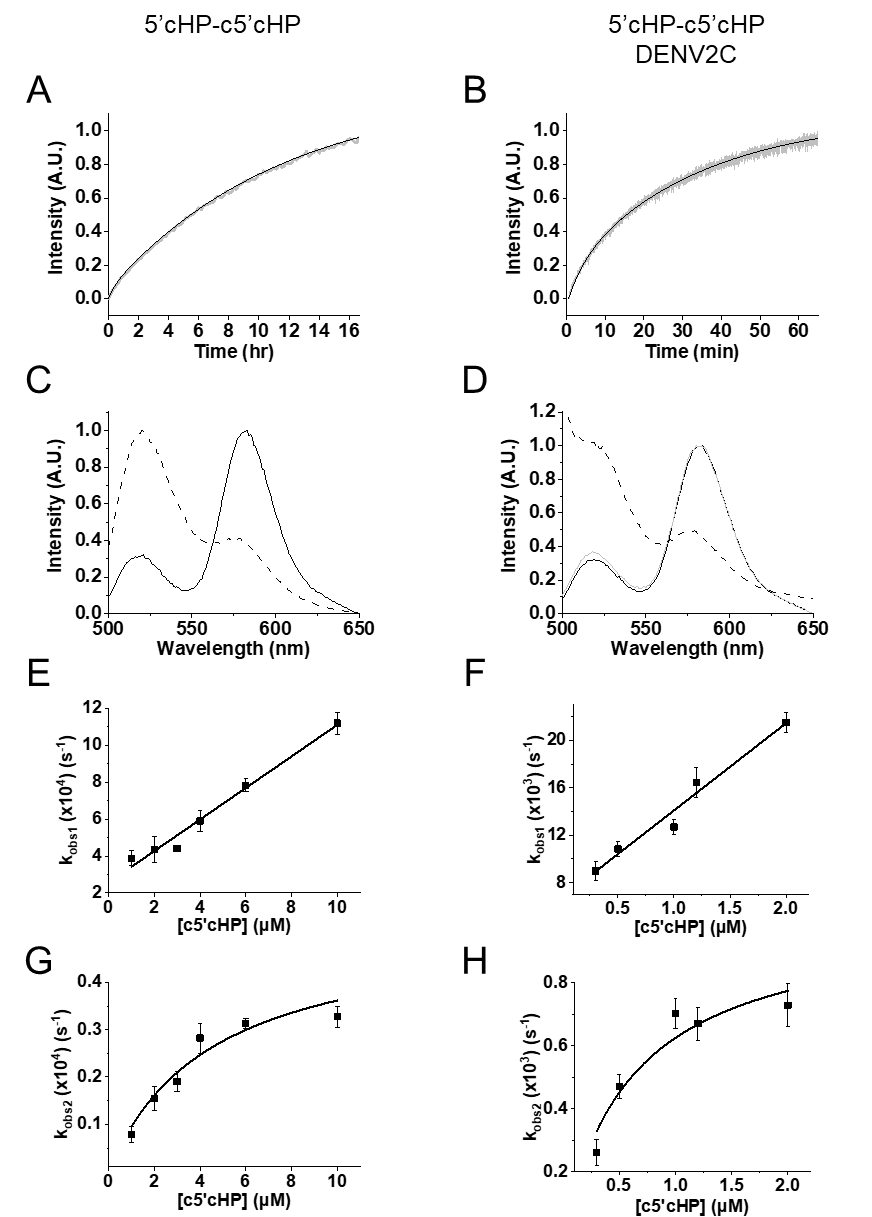


**Figure S4 – Real-time progress intensity curves (A-B), emission spectra (C-D) and kinetic parameters (E-H) of 5’cHP/c5’cHP annealing in the absence (A, C, E and G) and presence (B, D, F and H) of DENV2C.** (A and B) Real-time intensity traces (grey) and their fits to Equation (1) (black) to obtain fast (*k*_obs1_) and slow (*k*_obs2_) kinetic parameters. Real-time intensity traces of (A) 10 nM 5’cHP with 10 μM c5’cHP and (B) 10 nM 5’cHP with 1 μM c5’cHP in the presence of DENV2C in a protein:ORN molar ratio of 2:1. (C and D) Emission spectra of 5’cHP before (solid black) and after annealing (dotted black) to c5’cHP, at excitation wavelength 480 nm. The solid grey line shows the emission spectra of 5’cHP after addition of DENV2C in a protein:ORN molar ratio of 2:1. *k*_obs1_ (E and F) and *k*_obs2_ (G and H) parameters were plotted against the concentration of c5’cHP and fitted to Equation (2) and (3), respectively.


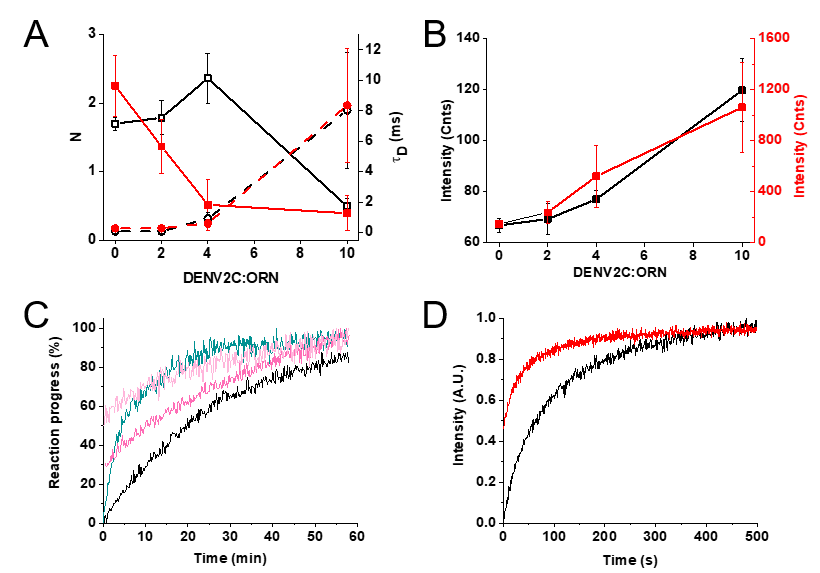


**Figure S5 – The optimal DENV2C:ORN molar ratio to use for annealing reactions is 2:1.** (A) Fluorescence correlation spectroscopy (FCS) was used to monitor aggregation of 10 nM 5’UAR RNA (black; open markers) or 10 nM DENV2C S24C mutant labeled with Atto488 dye (red; solid markers) at various DENV2C:ORN concentrations (0, 2:1, 4:1 and 10:1). The diffusion time, τ_D_ (circles; dotted line) and number of particles, N (squares; solid line) were measured. DENV2C S24C was generated by replacing serine at position 24 with a cysteine that allows labelling with Atto488. DENV2C S24C protein has a similar annealing activity as the wild-type protein, determined by comparing the observed kinetic values obtained from the annealing reaction between 10 nM 5’UAR and 500 nM c5’UAR in the presence of wild-type (k_obs1_ and k_obs2_ of 1.49 × 10^‑2^ s^‑1^ and 2.13 × 10^-3^ s^-1^) or mutant DENV2C (k_obs1_ and k_obs2_ of 1.54 × 10^-2^ s^-1^ and 2.32 × 10^-3^ s^-1^) at a DENV2C:ORN molar ratio of 2:1. A 543 nm continuous wave laser was used, with a 600/50 bandpass filter to monitor fluorescence from 3’TAMRA on 5’UAR. A 485 nm pulsed (20 MHz) laser was used, with a 510/20 bandpass filter to monitor fluorescence from Atto488‑labeled DENV2C protein. Error bars show standard deviation from three measurements. A DENV2C:ORN ratio of 2:1 was chosen to avoid aggregation conditions which start at a DENV2C:ORN of 4:1. An increase in the values of the τ_D_ from 0.069 ± 0.003 μm^2^/s to 0.92 ± 0.38 μm^2^/s for RNA and from 0.28 ± 0.05 μm^2^/s to 0.61 ± 0.25 μm^2^/s for DENV2C was observed, indicating aggregation beyond a DENV2C:ORN ratio of 2:1. With aggregation, N also decreases by 3.6-folds and 3.5-folds for RNA and DENV2C respectively. (B) Maximum intensity of Atto488-labeled DENV2C (black squares; solid line) and 5’UAR (red square; solid line) observed during FCS at various DENV2C:ORN molar ratios. The intensity of fluorescent particles increased by 4.5-fold and ~2-folds for RNA and DENV2C, respectively with increase in DENV2:ORN ratio above 2:1, suggesting the nature of DENV2C and RNA aggregates. Our data indicates that DENV2C:ORN aggregates above ratio 2:1 possibly consist of multiple RNAs complexed with multiple protein molecules. (C) Dose-response plot for DENV2C using the annealing reaction between 10 nM 5’UAR and 300 nM c5’UAR at DENV2C:ORN ratios of 2:1 (black), 4:1 (green), 10:1 (magenta) and 20:1 (light pink). Fitting of the intensity curve using Equation (1) provided observed kinetic values for the 5’UAR/c5’UAR reaction at DENV2C:ORN of 2:1 (k_obs1_ and k_obs2_ of 5.98 × 10^-3^ s^-1^ and 3.11 × 10^-4^ s^-1^) ,4:1 (k_obs1_ and k_obs2_ of 8.33 × 10^-3^ s^-1^ and 1.39 × 10^-3^ s^-1^) and 10:1 (k_obs1_ and k_obs2_ of 3.93 × 10^-2^ s^-1^ and 3.44 × 10^-4^ s^-1^). It was observed that ~30-50% annealing reaction is completed even before measurement. Thus, reliable and reproducible data could not be extracted via fitting using Equation (1) at higher DENV2C:ORN ratio, at least in our experimental conditions. (D) Intensity trace of annealing reaction between 10 nM 5’UAR and 500 nM c5’UAR in the presence of DENV2C, at a DENV2C:ORN molar ratio of 2:1 at 20^o^C (black) and 40^o^C (red). Fitting of the intensity curves using Equation (1) provided observed kinetic values for these reactions at 20^o^C (k_obs1_ and k_obs2_ of 1.54 × 10^-2^ s^-1^ and 2.04 × 10^-3^ s^-1^) and 40^o^C (k_obs1_ and k_obs2_ of 0.36 s^-1^ and 1.52 × 10^-2^ s^-1^). At higher temperatures, reliable and reproducible data could not be extracted as ~50% of the annealing reaction has finished at the initial point of measurement. Therefore, 20^o^C was chosen as the temperature to conduct all annealing reactions, unless otherwise stated. This was done to allow reliable fitting of intensity traces to obtain observed kinetic values for high concentrations of complementary sequences.


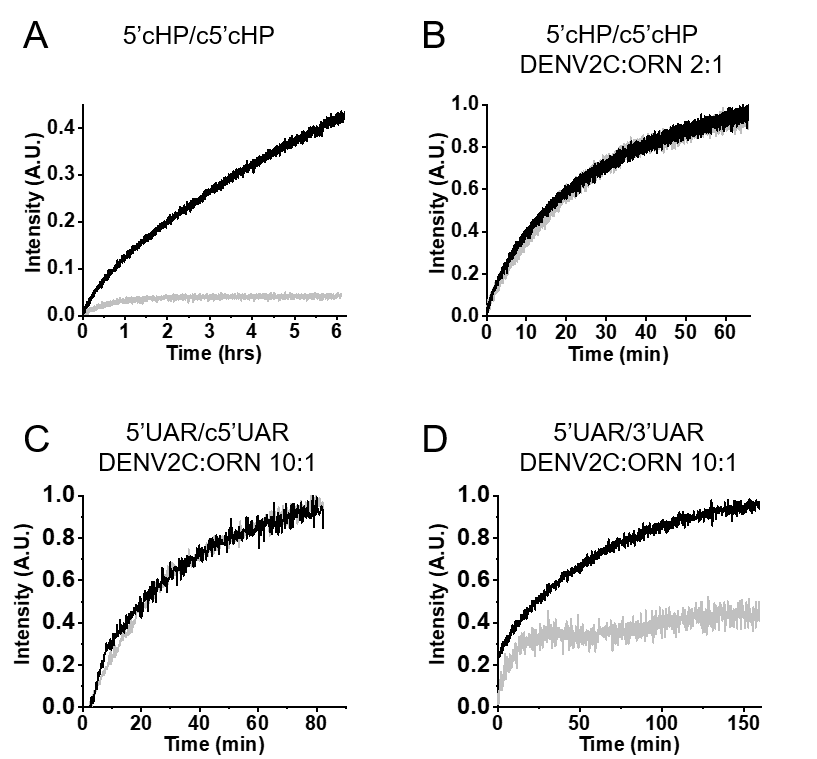


**Figure S6 – Real-time progress intensity traces of dual-labeled 5’cHP (black) or 5’cHP-ULoop mutant (grey) with its complementary sequence c5’cHP in the (A) absence and (B) presence of DENV2C.** (A) 5 μM c5’cHP and (B) 1 μM c5’cHP were used for the reaction with 5’cHP and 5’cHP-ULoop, respectively. (B) Fitting of the real-time intensity curves using Equation (1) provided observed kinetic values for the 5’cHP/c5’cHP reaction (*k*_obs1_ and *k*_obs2_ are 1.3 × 10^-2^ s^-1^ and 7.0 × 10^-4^ s^-1^) and the 5’cHP‑ULoop/c5’cHP reaction (*k*_obs1_ and *k*_obs2_ of 1.4 × 10^-2^ s^‑1^ and 6.8 × 10^-4^ s^-1^). **Reaction pathway of (C) 5’UAR/c5’UAR and (D) 5’UAR/3’UAR at a DENV2C:ORN molar ratio of 10:1.** Real-time intensity traces of 10 nM 5’UAR (black) or 5’UAR-ULoop mutant (grey) with its complementary sequences (C) 300 nM c5’UAR and (D) 500 nM 3’UAR at a DENV2C:ORN molar ratio of 10:1. (C) Fitting of the intensity curve using Equation (1) provided observed kinetic values for the 5’UAR/c5’UAR reaction (*k*_obs1_ and *k*_obs2_ of 3.12 × 10^-2^ s^-1^ and 5.34 × 10^-4^ s^-1^) and the 5’UAR-ULoop/c5’UAR reaction (*k*_obs1_ and *k*_obs2_ of 3.36 × 10^‑2^ s^‑1^ and 5.46  × 10^-4^ s^-1^). Excitation and emission wavelengths used were 480 nm and 520 nm respectively. For reactions performed in the presence of DENV2C, a DENV2C:ORN molar ratio of 2:1 was used. The result suggests that the annealing mechanism of 5’UAR/c5’UAR still proceeds predominantly through stem-stem interactions. At aggregating conditions, the annealing of 5’UAR‑ULoop mutant with 3’UAR is still slower than that of the wild-type, only reaching ~40% reaction completion when the 5’UAR/3’UAR reaction has completed. Although the reaction involving the ULoop mutant is clearly slower than the wild-type 5’UAR, the reaction at aggregating condition has clearly progressed further (40%) than the reaction at non-aggregating conditions (13%) (Fig. 5C). At aggregation conditions of DENV2C:ORN molar ratio of 10:1, the annealing mechanism of 5’UAR/3’UAR is still predominantly through kissing-loop interactions.

**References**

[1] M.N. Vo, G. Barany, I. Rouzina, K. Musier-Forsyth, Mechanistic Studies of Mini-TAR RNA/DNA Annealing in the Absence and Presence of HIV-1 Nucleocapsid Protein, J. Mol. Biol. 363 (2006) 244–261. https://doi.org/10.1016/j.jmb.2006.08.039.

[2] M.N. Vo, G. Barany, I. Rouzina, K. Musier-Forsyth, HIV-1 Nucleocapsid Protein Switches the Pathway of Transactivation Response Element RNA/DNA Annealing from Loop-Loop “Kissing” to “Zipper,” J. Mol. Biol. 386 (2009) 789–801. https://doi.org/10.1016/j.jmb.2008.12.070.

[3] C.R. Cantor, P.R. Schimmel, Biophysical chemistry: Part III: the behavior of biological macromolecules, Macmillan, 1980.
